# Clinical and radiographic outcomes after total hip arthroplasty with the NANOS neck preserving hip stem: a 10 to 16-year follow-up study

**DOI:** 10.1186/s12891-021-04953-8

**Published:** 2022-01-10

**Authors:** Vincenzo De Santis, Nadia Bonfiglio, Mattia Basilico, Greta Tanzi Germani, Maria Rosaria Matrangolo, Angelo Carosini, Giuseppe Malerba, Giulio Maccauro

**Affiliations:** 1 Department of Orthopaedics, Mater Olbia Hospital, Olbia, Italy; 2 Università Cattolica del Sacro Cuore, Rome, Italy; 3 Department of Orthopaedics, Fondazione Policlinico Universitario A. Gemelli IRCSS, Università Cattolica del Sacro Cuore, Largo A. Gemelli 8, 00168 Rome, RM Italy

**Keywords:** Total hip arthroplasty, Short stem hip arthroplasty, Survivorship, Outcome

## Abstract

**Background:**

Short-stem Hip Arthroplasty (SHA) are increasingly implanted in recent years thanks to their potential advantage in preserving metaphyseal bone-stock. Among them, the NANOS® short-stem implant demonstrated satisfactory results to short and mid-term. The purpose of this retrospective study was to evaluate the clinical and radiographic outcome of the Nanos® short stem at a minimum follow-up of 10 years.

**Methods:**

Sixty-seven patients aged 53 ± 20 years were enlisted in the study, for a total of 72 hips. Primary outcomes were survivorship of the implant and clinical outcome measured using the Hip disability and Osteoarthritis Outcome scores (HOOS) and the Short Form Survey (SF12) questionnaire. The secondary outcome was a radiological evaluation calculating the inclination and the anteversion angle of the acetabular cup for each implant and investigating osteolysis, heterotopic ossifications and stem position.

**Results:**

We observed a 95.5% stem survivorship. The complication rate was 7.6% and three implants underwent revision because of an aseptic loosening, an infection and a periprosthetic fracture due to trauma.

Among 58 patients (63 hips) evaluated in an outpatient visit 10–16 years after surgery, improvement in clinically relevant scores comparing with baseline was observed: HOOS score increased after surgery in all its subcategories (from 32.25 ± 14.07% up to 91.91 ± 9.13%) as well as SF12 which increased by more than 18 percentage points.

On clinical assessment, the range of motion (ROM) was restored at follow-up, 1 patient (1.7%) showed a squeaking hip and 2 (3.4%) reported *leg-length discrepancy.* Neutral stem positioning was achieved in 58 hips and heterotopic ossifications occurred in 10 hips (16%).

**Conclusions:**

The current study reports good clinical and radiological outcomes following NANOS® short-stem hip implant at minimum 10 years-follow-up. Since the high rate of stem survivorship, the low complication rate demonstrated and the overall patient satisfaction, our results suggest NANOS® neck-preserving prostheses should be considered as a valid alternative to standard implants.

## Background

The incidence of Total Hip Arthroplasty (THA) in young patients is currently increasing [1]. Restoring high-impact lifestyle and attaining high levels of pain relief and enhanced function are the targets after THA in young patients [2]. Nevertheless, it involves a greater risk of long-term complications and therefore future revision within their lifetime [3]. As a result, it was necessary to guarantee a longer life of the implants, introducing more wear resistant bearing surfaces and developing prosthetic models that preserve the bone stock [4–6]. The amount of remaining bone stock is an important technical aspect in planning revision hip arthroplasty [7].

The short-stem implants were designed to facilitate a minimally invasive approach since they anchored mostly in the femoral head-metaphysis area and were hypothesized to preserve more bone stock ensuring better condition in case of revision surgery [8–15]. Several Short-stem Hip Arthroplasty (SHA) implants have been introduced and developed during the last decades [16–18]. They are classified according to several items such as the anchoring femoral zone, the anatomic region they occupy, main stress distribution zones, geometric design, bone resection level in the femoral head, neck or metaphysis and the orientation axes used for insertion [8, 17, 19, 20]. Though long-term studies are not available yet, the SHAs have demonstrated good short- and mid-term clinical and radiographic outcomes and early survival rates comparable to standard stems [21–25].

Among them, the NANOS® short-stem implant (Smith & Nephew, Marl, Germany) demonstrated satisfactory results to short- and mid-term [23, 26–28]. The NANOS® short-stem implant was introduced in 2004 and it is designed to have an extended contact area at the calcar region to ensure optimal load transfer and to bind along the distal lateral cortex to support and compensate for varus load [11]. The NANOS® stem is made of a proximal osteoconductive coated titanium forged-alloy (ISO 5832-3) and an additional calcium phosphate coating. It is a partial collum design that yields metaphyseal anchoring and load transfer and requires minimal bone resection [29]. Since a minimum of ten-year survival should be considered to support the comparison with conventional stems, this retrospective study aims to evaluate the survivorship and the clinical outcome and secondly the radiographic outcome of NANOS® short-stem prosthesis over 10 years of follow-up [30]. We assume that NANOS neck preserving stems are a viable option in hip arthroplasty, particularly in young patients.

## Material and methods

Through our hospital database system, we identified a cohort of patients who underwent total hip replacement using the NANOS® short-stem prosthesis between 2005, when the implant was introduced in our hospital, and January 2011, in order to have a minimum follow-up of 10 years.

The inclusion criteria were: patients undergoing THA from 2005 to January 2011 performed by the same surgeon G.M.L.; the same stem NANOS® used for all subjects; expression of informed consent to take part in the study.

The patient’s medical records and the operative reports were reviewed in order to collect clinical scores and surgery data of each subject.

Stem survivorship and a clinical assessment were considered as the primary outcomes.

The secondary outcome was the radiographic assessment.

In January 2021 the patients were called to confirm the vital status. The available subjects were evaluated in an outpatient visit and questioned about any complications that had occurred.

### Surgical technique

NANOS® stem (sizes 2–6) was implanted in combination with a ceramic femoral head and insert (BIOLOX forte, Rotkreuz, Switzerland), and EP-FIT PLUS® (Smith and Nephew, Rotkreuz, Switzerland) acetabular cup.

Thirty minutes before incision, a single preoperative dose (2 g) of cefazolin was used as antimicrobial prophylaxis.

All implantations were performed with the patient in the lateral position, using the Gibson-Moore posterolateral approach.

The insertion of the short external rotator muscles was sectioned and then reinserted at the end of the implantation.

The femoral neck resection was done with a straight cross-cut osteotomy, preserving maximum bone stock. After acetabular cup implantation, the femoral path was prepared by the opening rasp and then by the forming rasps that were inserted with a slightly curved motion. This was followed by finishing with the cancellous compactors.

Then the trial positioning took place and the range of motion and leg length were checked.

Finally, NANOS® stem, ceramic femoral head and ceramic insert were implanted. The wound was sutured.

### Post-surgery routine

No intra-articular drainage was positioned after surgery; all patients wore graduated compression stockings postoperatively [31]. Mobilization and physiotherapy began the day after surgery; all patients were allowed to fully weight-bear with crutches, except for one patient who was allowed to protect weight-bearing for 15 days due to an intraoperative incomplete fracture. No antibiotic was administered post-surgery. All subjects received low-molecular-weight Heparin (LMWHs) once a day for 5 weeks postoperatively as antithrombotic prophylaxis.

### Clinical and radiographic evaluation

Survivorship analysis was performed considering revision as the endpoint.

In outpatient visit the patients were clinically and radiographically evaluated by N.B. and G.T.G.. Any discordance was solved by consensus with a third senior author (G.M.)

The clinical examination consisted of the evaluation of the hip range of motion, leg-length discrepancy (LLD) which has been measured by the method “anterior superior iliac spine (ASIS) to the medial malleolus”, with the patient lying on the examination table [32], presence of hip pain assessed with VAS scale, or any evocable audible noises of the hip as squeaking, clicking and grinding. Squeaking has been considered as a high-pitched audible sound from the hip; clicking as a “click” that occurs during hip movement or walking; grinding as “crepitus” during movement [33].

Two questionnaires were administered to each subject: the Hip disability and Osteoarthritis Outcome Score (HOOS) and the Short Form health survey (SF-12). They are both self-reported questionnaires.

The HOOS evaluates the assessment of the hip by analyzing 5 items: pain, symptoms, activities of daily living, sport and hip-related quality of life; the higher percentage value, the higher self-satisfaction [34, 35]. The SF-12 score evaluates physical and mental health; a lower score corresponds to a higher disability [36, 37].

The postoperative scores were then compared to the preoperative ones.

The radiographic evaluation was based on an AP X-ray of the pelvis in order to calculate hip socket inclination and anteversion, investigate presence of osteolysis, heterotopic ossifications and evaluate stem position.

**Fig. 1:**
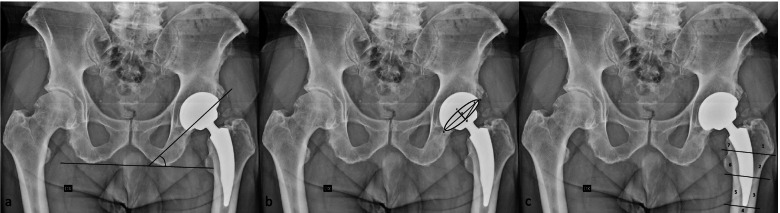
Radiographic evaluation. Techniques of measurement of the inclination acetabular cup (**a**), ante-version acetabular cup following method explained by Bachal et al. [37] (**b**) and osteolysis areas according to the Gruen zones (**c**)

The cup inclination was measured on standard X-ray as the angle between a line drawn along the opening of the acetabular component and a line joining the ischial tuberosities (Fig. 1a) while the anteversion was calculated according to Bachal’s method (Fig. 1b) [38, 39].

Any areas of osteolysis were investigated according to the Gruen zones (Fig. 1c), and any heterotopic ossifications following the Brooker Classification System [40, 41].

Stem position was considered varus or valgus when the tip of the stem touched the medial or lateral cortex, respectively [26].

## Results

Between March 2005 and January 2011, 67 patients underwent total hip arthroplasty using the NANOS® short-stem were recruited. Since 5 patients had bilateral arthroplasty, the total hips were 72 (32 left and 40 right hips).

Forty-three patients were males and 24 were females, with a mean age of 53 years (range 20–72 years). The mean follow-up was 13.3 years (10–16 years).

The diagnoses were primary osteoarthritis (82%), femoral head avascular necrosis (FHAN) (7%), post-traumatic osteoarthritis (11%) (Table 1).

**Table 1:** Patient characteristics

Patients	67
THA	72
Patients with bilateral THA (%)	5 (7.5%)
Men	43
Women	24
Age (y), mean ± SD	53 ± 20
**Ethiologies**	
Primary osteoarthritis (%)	82%
FHAN (%)	7%
Post-traumatic osteoarthritis (%)	11%
**Complications**	
Asepting loosening	1
Infection	1
Periprosthetic fracture	1
Intraoperative fracture	1
Dislocation	1

*Abbreviations*: *THA* total hip arthroplasty, *FHAN* Femoral head avascular necrosis

The head size was 36 mm in 82% (*n* = 59 THA), 32 mm in 14% (*n* = 10 THA) and 28 mm in 4% of cases (*n* = 3 THA). The medium size of the stem was 3 (range 2–6).

The size of acetabular cup was different between men and women: in the first group the 50 mm cup was used in 10 implants (14%), 52 mm in 14 (19%) THA), 56 mm in 17 (23%) and 58 mm in 5 (7%) THA). In the second group 48 mm cup was implanted in 13 hips (19%), 50 mm in 7 (10%), and 46 mm in 6 (8%).

Six patients were lost to follow-up as four of them died and 2 did not answer the phone and were excluded from the analysis.

**Fig. 2:**
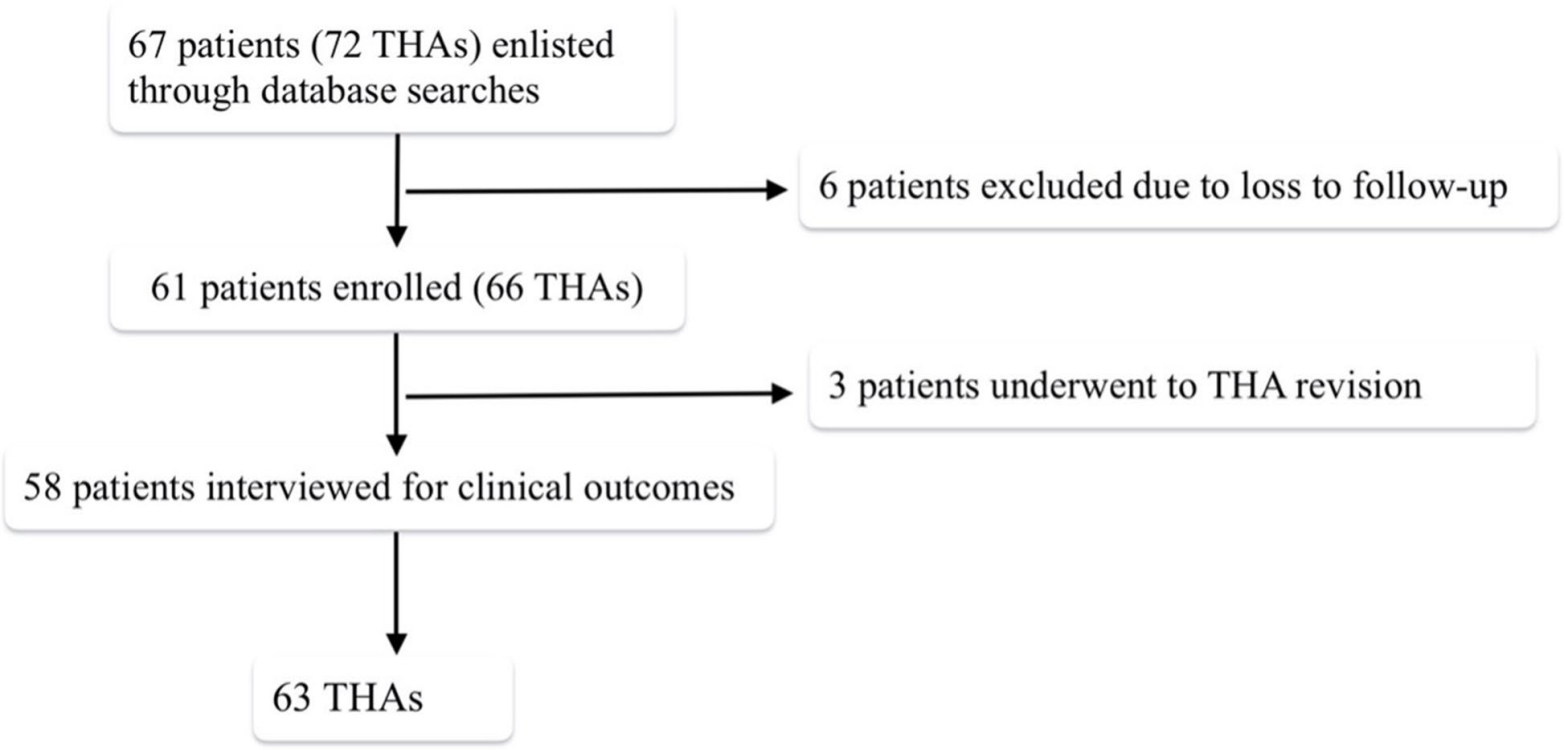
Inclusion process of patients. Flowchart of subject availability. *Abbreviations*: THA: total hip arthroplasty

Among the remaining 61 patients (66 hips), 5 (7.6%) implants had complications; 3 of these underwent revision, because of an aseptic loosening after 12 years (1.5%), an infection identified within 5 months (1.5%), a periprosthetic fracture 2 years after surgery (1.5%). They were included in the complication rate and in the stem survivorship rate but not considered in clinical and radiographic evaluation (Fig. 2).

The other complications were an intraoperative incomplete fracture (1.4%) of the calcar region of the femur that was synthesized with a circle band without implant sequelae and a dislocation occurred 2 months after surgery (1.4%) treated without surgery and then both were included in the outpatient evaluation.

Overall, the stem survivorship was 95.5%.

Finally, 58 patients were evaluated in an outpatient visit and questioned about any complications that had occurred after surgery. Five of them had bilateral prostheses, so a total of 63 cementless SHAa were evaluated clinically and radiographically.

### Clinical results

On clinical observation, leg-length discrepancy after surgery was reported in two patients (3.4%), but none of these exceeded 15 mm or was painful and 1 squeaking hip was found (1.6%). The range of motion (ROM) for extension/flexion, internal rotation/external rotation were restored at follow-up, none of the patients had a Trendelenburg gait or reported severe or disabling pain, as shown by the score of the visual analog scale (VAS).

A clinical and functional improvement was observed by HOOS and SF12 scores. We analyzed the Hip disability and Osteoarthritis Outcome Score (HOOS) and the Short Form Survey (SF-12) comparing the data before surgery (extrapolated from medical records) and after surgery. The mean HOOS score increased from 32.25 ± 14.07% preoperatively to 91.91 ± 9.13% at the final follow-up, with an increase of 60 percentage points. In addition, each subcategory of the HOOS questionnaire showed an improvement at follow-up, as demonstrated by preoperative and postoperative mean value (expressed in percentage points) and their relative difference (Δ):

32.94 ± 15.69% and 89.68 ± 12.14% for the S-section (Symptoms & Stiffness) (Δ = 56.74 points);

33.25 ± 18.80% and 94.37 ± 7.28% for the P-section (Pain) (Δ = 61.12 points);

33.29 ± 14.45% and 80.16 ± 8.19% for the A-section (Function, daily living) (Δ = 46.87 points);

22.04 ± 17.95% and 85.17 ± 13.65% for the SP-section (Function, sports and recreational activities) (Δ = 62.77 points);

and, finally, 19.94 ± 16.51% and 74.42 ± 19.03% for the Q-section (Quality of life) (Δ = 54.48 points).

**Fig. 3:**
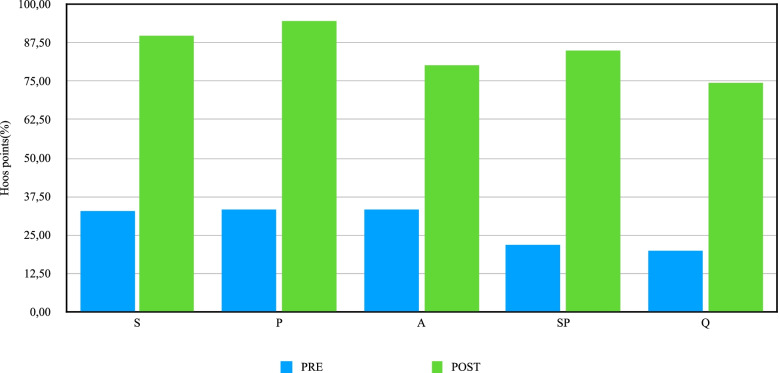
Clinical outcome**.** HOOS subcategories scores before and after surgery. *Abbreviations*: HOOS = Hip Disability and Osteoarthritis Outcome; S: Symptoms & Stiffness section; P: Pain section; A: Function, daily living section; SP: Function, sports and recreational activities section, Q: Quality of life section

An important increase was also achieved in SF12 questionnaire, starting from a score of 28.57 ± 9.93 to 47.09% ± 5.96 for the PCS (Physical Score) section (Δ = 18.07) and 36.08 ± 9.14% to 54.15 ± 8.52% for MCS (Mental Score) section (Δ = 18.52) (Table 2 & Fig. 3.)

**Table 2:** Clinical scores

	Subcategories	Preoperative	Follow-up at 10–16 years	Δ (points)
**HOOS total score (0–100)**		32.25% (σ = 14.07)	91.91% (σ = 9.13)	59.66
	S (Symptoms & Stiffness)	32.94% (σ = 15.69)	89.68% (σ = 12.14)	56.74
	P (Pain)	33.25% (σ = 18.80)	94.37% (σ = 7.28)	61.12
	A (Function, daily living)	33.29% (σ = 14.45)	80.16% (σ = 8.19)	46.87
	SP (Function, sports and recreational activities)	22.04% (σ = 17.95)	85.17% (σ = 13.65)	62.77
	Q (Quality of life)	19.94% (σ = 16.51)	74.42% (σ = 19.03)	54.48
**SF12 PCS score**		36.08% (σ = 9.14)	54.15% (σ = 8.52)	18.07
**SF12 MCS score**		28.57% (σ = 9.93)	47.09% (σ = 5.96)	18.52

Clinical outcome of patients treated with the Nanos total hip implant, assessed through Hip disability and Osteoarthritis Outcome Score (HOOS) and Short Form Survey (SF-12) *Abbreviations*: *HOOS* Hip Disability and Osteoarthritis Outcome, *SF12* Short Form Survey, *PCS* Physical score, *MCS* Mental score, standard deviation

### Radiological results

**Fig. 4:**
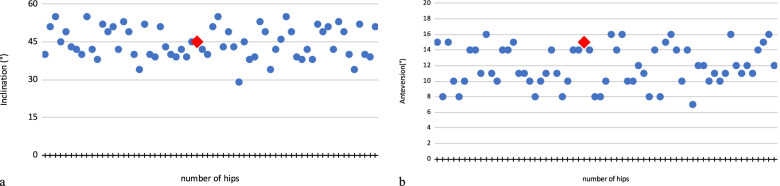
Acetabular cup inclination and anteversion angles. Figures show degree of inclination (**a**) and anteversion (**b**) for each hip in comparison to 45° of inclination and 15° of anteversion (point red), considered the desired target

On radiographs a total of 63 hips were assessed; the mean acetabular inclination was 45° (range 38°- 55°) and the mean value of the ante-version angle was 12° (range 15° ± 10°) (Fig. 4).

Three osteolysis were identified in zone 1 e one in zone 7 according to Gruen zone.

Heterotopic ossifications occurred in 10 hips (15%): 4 (6%) were classified as Brooker I, 4 (6%) as Brooker II, and 2 (3%) as Brooker III. Neutral stem positioning was achieved in 58 hips (92%) and 5 stems (8%) were valgus.

## Discussion

Our retrospective study showed interesting results concerning the NANOS neck preserving stems outcomes, at a minimum of 10 years of follow-up.

As the incidence of THA in young patients has increased, several short stem implants have been introduced to achieve minimally invasive surgery, faster rehabilitation and bone stoke sparing [1, 8, 11]. Preservation of proximal femoral bone is critical considering that the lower the average age, the greater the risk of revision. According to Bieger et al., the preservation of the bone stock should potentially reduce stress shielding and bone resorption thanks to better load distribution [42]. SHAs were introduced during the last decades so there are only few mid- and long-terms studies. To the authors’ knowledge, the current is the longest follow-up study of patients receiving the same single short-stem implants.

Our study showed good to excellent clinical results at a minimum of 10-years follow-up. Both HOOS and SF12 showed a significant improvement from preoperative to the last follow-up. No tight pain was identified, confirming a lower incidence of this symptom in SHAs than in conventional THAs as shown by Banerjee et al. [43]. One patient declared an occasional noisy sound from his hip replacement that we detected as a squeaking sound. Similar clinical results at 5.6 years of follow-up were reported by Capone et al., revealing NANOS® stem implant as a safe option even in osteonecrosis of the femoral head, for which traditional THA has resulted in worse outcomes than other indications [26]. The complication rate was 7.6% and the revision rate was 4.5% because of an aseptic loosening, an infection and a periprosthetic fracture due to trauma; one patient had a dislocation treated with closed reduction and one patient had an intraoperative fracture that was solved with a cerclage without any sequelae. According to the literature, the number of complications is comparable with those of conventional stems [44]. We observed a 95.5% stem survivorship. Other studies published higher survivorship rates for Nanos stems; no stem revisions were reported by Capone et al. and Ettinger et al. and only 2 (1.9%) revisions were identified by Budde et al. in their cohort after 24 months [23, 26, 27]. Although our results show lower survivorship rates, they are concerning a longer follow-up; in comparison with other short-stems and standard implants, similar survival rates are reported in the literature [45].

It is increasingly accepted that preserving bone stock is essential in case of revision surgery and should potentially reduce complications such as loosening of the stem or periprosthetic fracture due to less bone resorption [46]. Furthermore, short-stem implants have proven to be a safe and efficient procedure that has demonstrated good primary stability and osteointegration and therefore a low risk of aseptic loosening despite their smaller surface area and implant-bone interface [12, 28, 47, 48]. On the other hand, the surgical technique requires precision and experience to avoid misalignment, incorrect sizing of the stems and intraoperative fracture and is related with a steep learning curve [30–43]. In this regard, our case series supports it since all the implants were performed by the same surgeon. This represents one of the strengths of the study, as well as the long-term follow-up. Since the average follow-up over 10 years, the current study represents a strong value in supporting short-stem hip arthroplasty, mainly in young patients.

### Limits of the study

The major limitation of this study is that it is a retrospective analysis so there is a lack of information on any comorbidities or preoperative clinical conditions of the patients that may have influenced the post-surgery period and therefore the parameters analyzed. Another limitation is the low sample of patients; six subjects were lost at follow-up, so the survivorship rate and complication rate could be different than those reported. Lastly, since the aim of the current study was to describe the outcomes of the analyzed subjects, there is no comparison with a control group. We consider a comparison with conventional stems should be a focus for future studies.

## Conclusions

This study shows that NANOS® short stem prostheses result in good clinical and radiological outcomes after 10-year minimum follow-up. Our results confirm the ones previously reported in shorter follow-up studies. We observed a high rate of stem survivorship and a low complication rate according to the literature, as well as high patient satisfaction. Despite the limits of the study, the results are promising and demonstrate that NANOS® neck-preserving prostheses represent a valid alternative to standard implants, mainly in young patients. Further prospective studies are needed with a larger sample to obtain ever more complete information.

## Data Availability

The datasets used and/or analyzed during the current study are available from the corresponding author on reasonable request.

## Abbreviations

SHA: Short-stem Hip Arthroplasty
THA: Total Hip Arthroplasty
HOOS: Hip disability and Osteoarthritis Outcome scores
SF12: Short Form Survey
LMWH: Low-molecular-weight Heparin
LLD: Leg-length discrepancy
ASIS: Anterior superior iliac spine
ROM: Range of motion
VAS: Visual analog scale
FHAN: Femoral head avascular necrosis
